# Effective screening of T cells recognizing neoantigens and construction of T-cell receptor-engineered T cells

**DOI:** 10.18632/oncotarget.24232

**Published:** 2018-01-13

**Authors:** Taigo Kato, Tatsuo Matsuda, Yuji Ikeda, Jae-Hyun Park, Matthias Leisegang, Sachiko Yoshimura, Tetsuro Hikichi, Makiko Harada, Makda Zewde, Sho Sato, Kosei Hasegawa, Kazuma Kiyotani, Yusuke Nakamura

**Affiliations:** ^1^ Department of Medicine, The University of Chicago, Chicago, IL 60637, USA; ^2^ Institute of Immunology – Campus Buch, Charité – Universitätsmedizin Berlin, Berlin 13125, Germany; ^3^ Berlin Institute of Health, Berlin 10117, Germany; ^4^ OncoTherapy Science Inc., Kawasaki, Kanagawa 213-0012, Japan; ^5^ Department of Gynecologic Oncology, Saitama Medical University International Medical Center, Hidaka, Saitama 350-1298, Japan; ^6^ Department of Surgery, The University of Chicago, Chicago, IL 60637, USA

**Keywords:** neoantigens, neoantigen-specific T cells, T cell receptor, TCR-engineered T cells

## Abstract

Neoantigens are the main targets of tumor-specific T cells reactivated by immune checkpoint-blocking antibodies or when using tumor-infiltrating T cells for adoptive therapy. While cancers often accumulate hundreds of mutations and harbor several immunogenic neoantigens, the repertoire of mutation-specific T cells in patients might be restricted. To bypass suboptimal conditions, which impede the reactivation of existing T cells or the priming of neoantigen-specific T cells in a patient, we employ T cells of healthy donors with an overlapping HLA repertoire to target cancer neoantigens. In this study, we focus on streamlining the process of *in vitro*-induction of neoantigen-specific T cells and isolating their T cell receptors (TCRs) to establish a time-efficient protocol that will allow the patient to benefit from subsequent therapy. We first optimized the priming of T cells to omit multiple restimulations and extended culturing. Neoantigen-specific T cells were enriched using specific dextramers and next-generation sequencing was applied to determine the TCR repertoire. This allowed us to circumvent the laborious process of expanding T cell clones. Using this protocol, we successfully identified HLA-A-restricted TCRs specific for neoantigens found in an esophageal cancer cell line (TE-8) and a primary ovarian cancer. To verify TCR specificity, we generated TCR-engineered T cells and confirmed recognition of the tumor-derived neoantigens. Our results also emphasize the importance of neoepitope selection in order to avoid cross-reactivity to corresponding wild-type peptide sequences. In conclusion, we established a 2-week protocol for generating and identifying neoantigen-specific TCRs from third-party donors making this strategy applicable for clinical use.

## INTRODUCTION

Cancer immunotherapies have revolutionized and improved cancer treatment through boosting the patients’ anti-tumor immune responses. Particularly, the success of immune checkpoint inhibitors has highlighted the importance of an effective anti-cancer immune response in cancer patients. However, the clinical outcome is still very limited and the majority of cancer patients has no benefit by these inhibitors [1, 2]. Therefore, it is important and urgent to clarify mechanisms that cause resistance and to develop a way to further enhance and improve the patients’ immune response [2].

Recently, several studies of cancer immunotherapy revealed that T cells recognizing cancer-specific antigens (neoantigens and shared antigens) may have significant impact on the clinical response. For instance, the higher numbers of somatic mutations may increase a chance to generate highly immunogenic neoantigens that induce/activate T cells, which are thought to drive the clinical effects of immune checkpoint inhibitors [1, 3–5]. In addition, programmed death-ligand 1 (PD-L1) expression on cancer cells was considered to reflect the activity of cancer-antigen specific T cells expressing PD-1 molecules; the interaction of these two molecules is in fact a target of PD-1/PD-L1 blockade [1, 6–8]. Furthermore, the success of adoptive therapy using autologous tumor-infiltrating lymphocytes (TILs) was correlated with the presence of T cells targeting cancer-specific antigens [9].

To enhance a patient’s T cell-mediated anti-tumor immune responses for further improvement of cancer immunotherapy, two approaches are currently investigated. First, peptide vaccines targeting cancer-specific antigens, neoantigens and shared antigens, can activate or induce antigen-specific T cells in cancer patients. As only neoantigens are non-self peptides derived from somatic non-synonymous mutations in cancer cells, they are potentially more immunogenic [10]. Given that the adoptive transfer of patient-derived neoantigen-specific T cells already showed positive clinical results [11, 12], the use of neoantigen vaccines might be an auspicious opportunity to induce or expand mutation-specific T cells in cancer patients [13]. However, recent studies suggest that patient-derived mutation-specific T cells might be functionally impaired because the cells are epigenetically imprinted to revert into an inactive state even after transient activation [14, 15]. This would favor a second approach using the transfer of cancer-specific T cell receptor (TCR) genes into T cells that can be isolated from the patients’ peripheral blood [16]. Preclinical studies have shown that the adoptive transfer of neoantigen-specific TCR-engineered T cells can be effective against large and long-established solid tumors [17, 18]. In order to render this strategy applicable for clinical use, we focused on refining a protocol that allows the effective and prompt identification of cancer neoantigen-specific TCRs using T cells derived from blood of human leucocyte antigen (HLA)-matched healthy donors [19]. Because the reactivation of existing T cells or the priming of neoantigen-specific T cells in a patient might be impaired, we propose this pipeline for the efficient generation of neoantigen-specific T cells and the identification of their TCRs to develop personalized and cancer-specific adoptive immunotherapies using TCR-engineered T cells especially for progressive tumor or for bulky tumor which needs urgent treatments.

## RESULTS

### A time-efficient protocol to prime and screen neoantitgen-specific T cells from third-party donors

**Figure 1:**
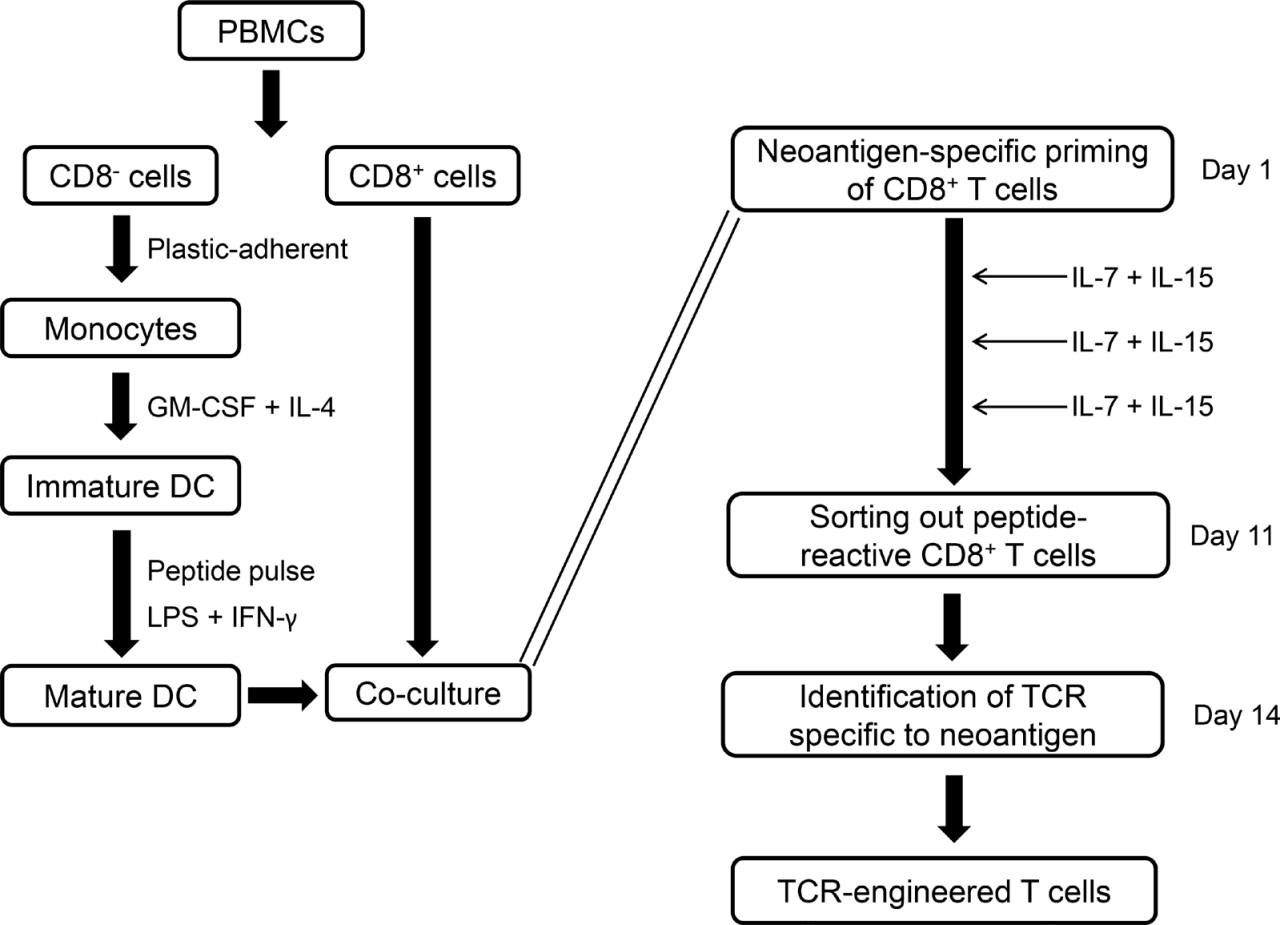
Screening of neoantigens which induce specific cytotoxic T lymphocytes (CTLs) Workflow of rapid screening protocol of neoantigen-reactive CTLs from donors’ blood. PBMCs: peripheral blood mononuclear cells, DCs: dendritic cells.

We first established the pipeline for the rapid identification of antigen-specific T cells from healthy donors’ peripheral blood mononuclear cells (PBMCs) to meet the purpose of clinical application using TCR-engineered T cells (Figure 1). After identification of candidate neoantigens by means of whole-exome and RNA sequencing, we prepared matured dendritic cells (DCs) loaded with neoantigen peptides as antigen presenting cells. To induce neoantigen-specific T cells, we initiated priming of autologous CD8^+^ T cells by incubation with peptide-pulsed DCs (day 1). Antigen-depending T cell stimulation was fostered by supplementing the culture with low amounts of IL-7 and IL-15 three days after the initial priming step (and subsequently every 2 days) [20]. Finally, the time period for inducing antigen-specific T cells was significantly shortened by harvesting the T cells already after the initial priming. Therefore, on day 11, neoantigen-specific CD8^+^ T cells were stained using peptide-HLA dextramers and we enriched the population by fluorescence activated cell sorting (FACS). Using RNAs from these sorted T cells, we completed TCR sequencing within next 3 days to identify TCR alpha (TCRA) and beta (TCRB) chains. Notably, the screening process to determine TCRA and TCRB genes of potentially neoantigen-specific TCRs was completed only within two weeks after initiating the priming of CD8^+^ T cells.

### Neoantigen-specific CD8^+^ T cells can be generated from HLA-matched third-party donors

To verify that the established protocol is suited to induce neoantigen-specific CD8^+^ T cells, we performed an analysis using the esophageal cancer cell line TE-8 and primary material obtained from an ovarian cancer patient. In our analyses, we focused either on HLA-A*24:02 (TE-8) or HLA-A*02:01 (ovarian cancer), which are the most common HLA class I types in either the Japanese or the Caucasian population, respectively.

**Figure 2:**
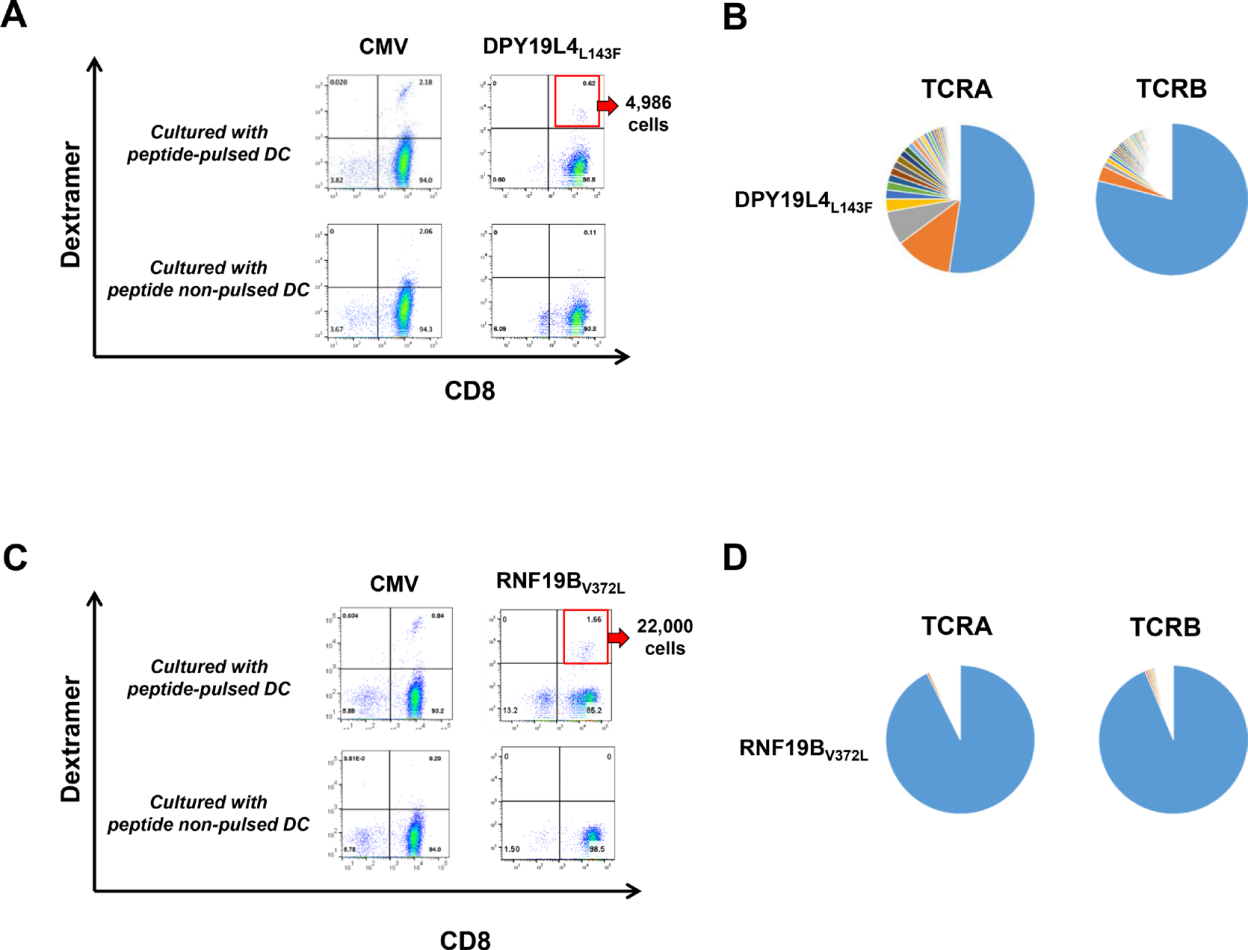
Identification of neoantigen-specific T cells using our rapid screening protocol (**A**) Peptide-HLA dextramer staining for CD8^+^ T cells co-cultured with autologous DCs with or without mutated peptide-pulse of DPY19L4_L143F_, which was identified in the COSMIC database. The number of sorted CD8^+^HLA-dextramer^+^ T cells was 4,986. (**B**) Frequency distributions of TCRA and TCRB sequences of CD8^+^ T cells reactive to DPY19L4_L143F_ peptide. After sorting the dextramer-positive cell population, TCR sequencing was performed. Each pie chart represents the frequency of unique TCRA and TCRB CDR3 sequences of sorted CD8^+^HLA-dextramer^+^ T cells. (**C**) Peptide-HLA dextramer staining for CD8^+^ T cells co-cultured with autologous DCs with or without mutated RNF19B_V372L_ peptide pulse, which was identified in an ovarian cancer patient. The number of sorted CD8^+^HLA-dextramer^+^ T cells was 22,000. (**D**) Frequency distributions of TCRA and TCRB sequences of CD8^+^ T cells reactive to RNF19B_V372L_ peptide. After sorting the dextramer-positive cell population, TCR sequencing was performed. Each pie chart represents the frequency of unique TCRA and TCRB CDR3 sequences of sorted CD8^+^HLA-dextramer^+^ T cells. Antigen peptide of CMV (cytomegalovirus) pp65 for HLA-A*24:02 or HLA-A*02:01 were used as a positive control.

In TE-8, we identified a total number of 312 non-synonymous mutations (Supplementary Figure 1). Although irrelevant for this exemplary analysis, it should be noted that no germline control was available for this established cell line and we were therefore unable to distinguish somatic mutations from rare germline variations. Subsequently, using a list of these possible mutations, we predicted the binding affinity of peptides including amino acid substitutions to a HLA-A2402 molecule and identified 81 neoantigen epitopes which showed IC_50_ of 500 nM or lower. We then confirmed the expression of RNA corresponding to 33 neoantigen epitopes with RNA sequencing data (Supplementary Table 1). This number of neoantigen epitopes was further narrowed to those showing an IC_50_ of 50 nM or lower to select peptides that were used to test induction of neoantigen-specific CD8^+^ T cells using peripheral blood from healthy donors. After priming of CD8^+^ T cells, we detected neoantigen-specific CD8^+^ T cells by staining with HLA-dextramers loaded with a neoantigen peptide derived from the missense substitution L143F in dpy-19 like 4 gene (DPY19L4_L143F,_ LYPEFIASI, Figure 2A). The proportion of CD8^+^HLA-dextramer^+^ T cells was 0.62% (4,986 cells). We subsequently performed TCR sequencing and determined the entire sequences of TCRA and TCRB chains with highly variable complementarity determining region 3 (CDR3) sequences in these sorted T cells (Figure 2B).

As a second example with higher clinical relevance, we aimed at generating neoantigen-specific CD8^+^ T cells against a mutation in an ovarian cancer sample. The material was isolated from a 64 year-old patient diagnosed with stage 3C clear cell ovarian carcinoma. We found a total of 80 non-synonymous mutations and calculated the affinity of potential neoantigen epitopes to a HLA-A0201 molecule (Supplementary Tables 2, 3). Among 32 candidate epitope peptides that showed a peptide-HLA affinity of 500 nM or lower and were expressed in tumor cells, we narrowed down the candidate neoepitopes (IC_50_ of 50 nM or lower) for testing their ability to induce CD8^+^ T cells isolated from a healthy donor (Supplementary Table 3). We successfully identified CD8^+^ T cells which were primed against a neoantigen peptide derived from RNF19B_V372L_ (V372L mutation in ring finger protein 19B gene, MLIGIPVYV, Figure 2C). The proportion of CD8^+^HLA-dextramer^+^ T cells was 1.66% (22,000 cells). We subsequently analyzed their TCR repertoire (Figure 2D).

Interestingly, in both experiments, even a short expansion time of 11 days was sufficient to stimulate the outgrowth of single T cell clone which TCRA and TCRB sequences dominated the TCR repertoire (Supplementary Table 4). Both TCR pairs were molecularly cloned and used for further analysis.

### TCRs isolated from DPY19L4_L143F_-reactive T cells of third party donors are mutation-specific

**Figure 3:**
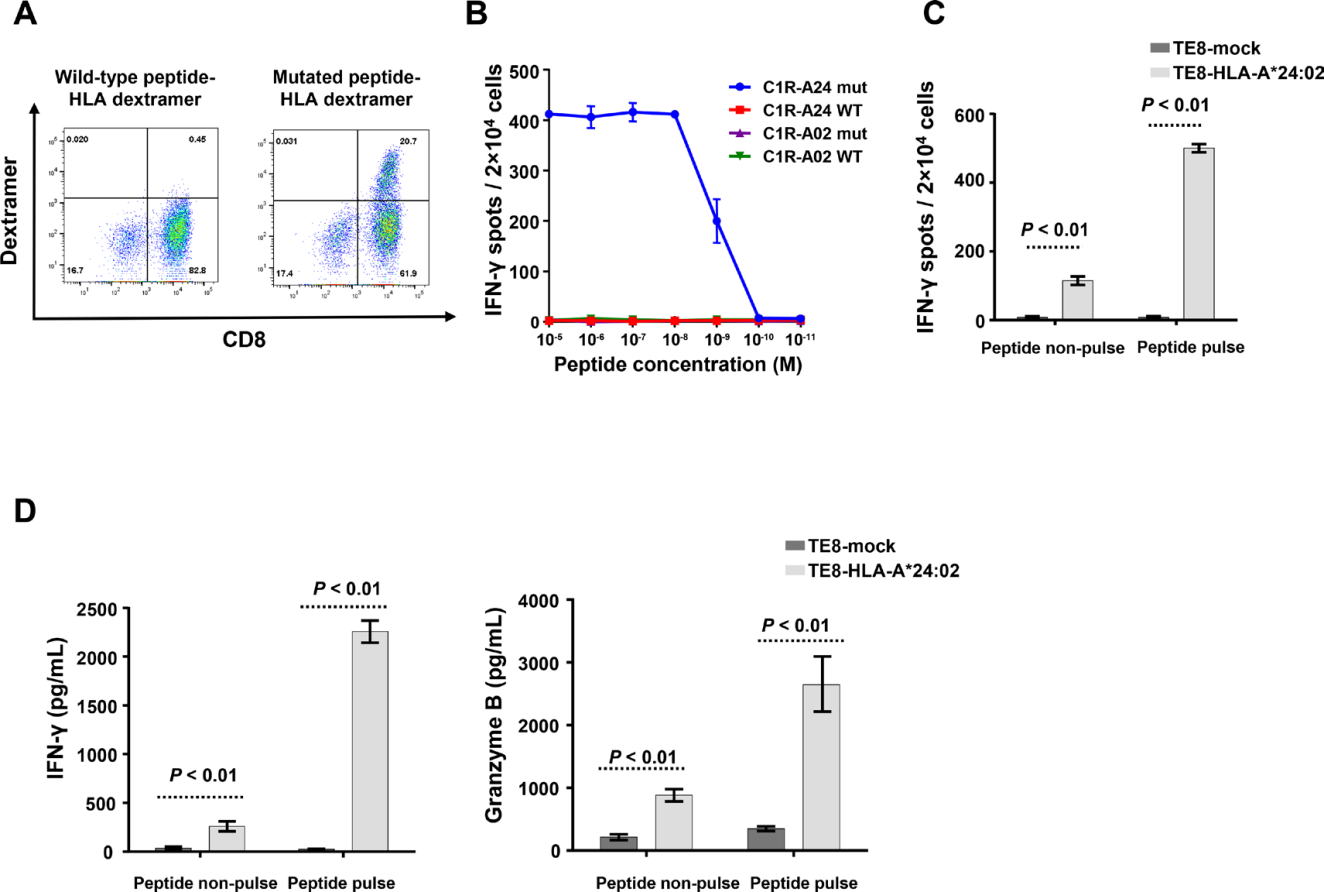
*In vitro* analysis of DPY19L4_L143F_ TCR-engineered T cells (**A**) Flow cytometric analysis of HLA-A*24:02 dextramer with wild-type or mutated peptide on DPY19L4_L143F_ TCR-engineered T cells. (**B**) IFN-γ ELISPOT assay on DPY19L4_L143F_ TCR-engineered T cells co-cultured with C1R-A24/A02 cells loaded with graded amounts of peptide. (**C**) IFN-γ ELISPOT assay on DPY19L4_L143F_ TCR-engineered T cells co-cultured with HLA-A*24:02- or mock-transfected TE-8 cells. (**D**) ELISA assays for IFN-γ, and granzyme B on DPY19L4_L143F_ TCR-engineered T cells co-cultured with HLA-A*24:02- or mock-transfected TE-8 cells.

Next, we generated a retroviral vector encoding for the DPY19L4_L143F_-TCR genes and transduced T cells derived from peripheral blood of a healthy donor (DPY19L4_L143F_ TCR-engineered T cells). DPY19L4_L143F_ TCR-engineered T cells showed binding of specific dextramers loaded with the mutant but not with the wild-type DPY19L4 peptide (Figure 3A). Furthermore, we confirmed antigen-specific function of DPY19L4_L143F_ TCR-engineered T cells by co-culture with C1R lymphoblastoid cells stably expressing either HLA-A*24:02 or HLA-A*02:01 and loaded with titrated amounts of either mutant or wild-type DPY19L4 peptide. Activation of TCR-engineered T cells was determined by measuring IFN-γ secretion using ELISPOT (Figure 3B) or ELISA (Supplementary Figure 2A) and by analyzing CD137 upregulation by FACS (Supplementary Figure 2B). The functional characterization substantiated the exquisite specificity of the isolated DPY19L4_L143F_-TCR because C1R cells were only recognized when (i) expressing HLA-A*24:02 and (ii) loaded with the mutant DPY19L4 peptide. The wild-type DPY19L4 peptide and HLA-A*02:01 were not sufficient to stimulate function of TCR-engineered T cells.

To test whether endogenously processed antigen can be recognized, we incubated DPY19L4_L143F_ TCR-engineered T cells together with TE-8 cancer cells that were reported to express the HLA-A*24:02 allele [21]. However, HLA expression could not be verified by FACS and surface presentation of endogenously processed DPY19L4_L143F_ antigen had to be restored by transfection of TE-8 cancer cells with an HLA-A*24:02 vector (Supplementary Figure 3). Hence, DPY19L4_L143F_ TCR-engineered T cells secreted IFN-γ only when incubated with HLA-A*24:02-transfected TE-8 cells, whereas mock-transfected TE-8 cells could not trigger T cell activation (Figure 3C, 3D). The TCR-engineered T cells also secreted the cytolytic molecule granzyme B (Figure 3D). In addition, when we pulsed HLA-A*24:02-transfected TE-8 cells with the mutant peptide, IFN-γ and granzyme B secretion was further enhanced (Figure 3C, 3D). These results indicate that DPY19L4_L143F_ TCR-engineered T cells recognized the endogenously-expressed mutated peptide in the HLA-A2402-restricted manner and showed cytotoxic activity.

To further explore the cytotoxic activity of T cells engineered with the DPY19L4_L143F_-TCR, we made use of HLA-A*24:02-positive TE-11 esophageal cancer cells since we could not establish TE-8 cells that stably express HLA-A*24:02 (Supplementary Figure 3). Direct killing of TE-11 cancer cells was only observed after loading with DPY19L4_L143F_ peptide (cell viability was reduced to 27.5%, Supplementary Movie 1). The cell viability of TE-11 cancer cells that were not loaded with peptide was only marginally impaired (reduced to 73.1%, Supplementary Movie 2).

### TCRs isolated from RNF19B_V372L_-reactive T cells recognizes the neoantigen peptide and its wild-type analog

**Figure 4:**
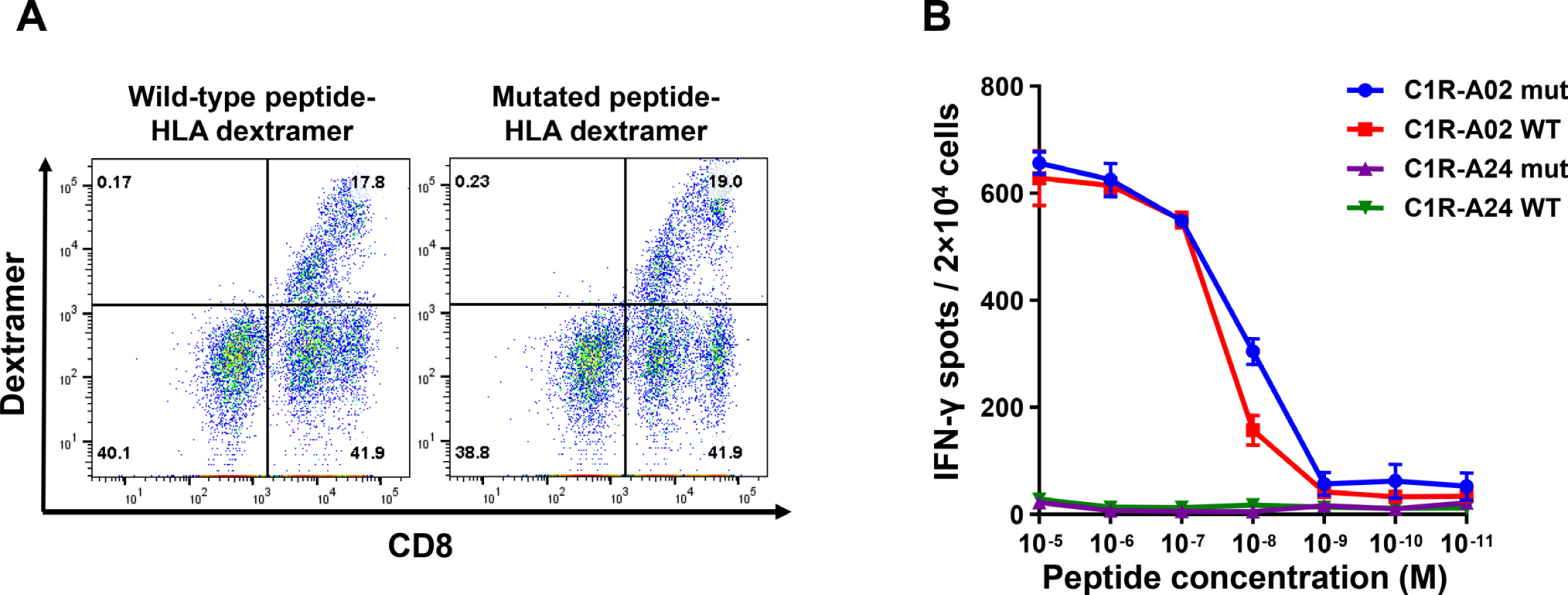
RNF19B_V372L_ TCR-engineered T cells cross-react towards the wild-type peptide (**A**) Flow cytometric analysis of HLA-A*02:01 dextramer with wild-type or mutated peptide on RNF19B_V372L_ TCR-engineered T cells. (**B**) IFN-γ ELISPOT assay on RNF19B_V372L_ TCR-engineered T cells co-cultured with C1R-A24/A02 cells loaded with graded amounts of peptide.

To analyze the TCR chains that were identified after priming of T cells against the RNF19B_V372L_ mutation, we constructed a retroviral vector encoding the RNF19B_V372L_-TCR genes and generated TCR-engineered T cells (RNF19B_V372L_ TCR-engineered T cells). In contrast to the analysis of the DPY19L4_L143F_-TCR, RNF19B_V372L_ TCR-engineered T cells bound dextramers irrespective of whether the HLAs were loaded with mutant or wild-type RNF19B_V372L_ peptide (Figure 4A). IFN-γ ELISPOT assay also revealed that RNF19B_V372L_ TCR-engineered T cells secreted IFN-γ at the similar levels when the antigen-presentation cells were pulsed with the wild-type and mutated peptides although the recognition of these peptides by RNF19B_V372L_ TCR-engineered T cells were confirmed to occur on an HLA-A0201-restricted manner (Figure 4B and Supplementary Figure 4). These results substantiate the potential risk that neoantigen-specific TCR-engineered T cells may be cross-reactive to the wild-type analog of neoantigen peptides and calls for judicious selection of neoantigen for T cell priming.

## DISCUSSION

Identification of human tumor antigens and immune checkpoint molecules significantly contributed to the better understanding of tumor immunology [22–24]. These findings were translated into the applied medicine, led to the development of effective immune checkpoint inhibitors, cancer peptide vaccine and adoptive cell transfer therapy (e.g. TIL infusion therapy) that have revolutionized cancer treatment [25–28]. In particular, several types of immune checkpoint inhibitor emerged as a novel cancer treatment after the first approval of a fully humanized antibody against cytotoxic T-lymphocyte-associated protein 4 (CTLA-4) for treatment of advanced melanoma and showed significant survival benefit in various types of cancer [2, 29]. However, recent meta-analysis of clinical data made it clear that only a subset of patients responded to immune checkpoint inhibitors, and the majority of patients had no benefit and some of them suffered from severe immune-related adverse reactions. Therefore, it is crucial to develop a new strategy to enhance the host anti-tumor immune response for further improvement of clinical outcomes in cancer immunotherapies.

In this study, we developed a time-efficient approach to identify neoantigen-specific TCRs that can be applied to neoantigen-specific TCR-engineered T cell therapy. Our approach has several major advantages. First, our protocol requires only two weeks in the process from the beginning of T cells priming with possible immunogenic neoantigen peptides to identification of neoantigen-specific TCRs (Figure 1). We previously established the protocol for *in vitro* induction of T cells specific to shared antigens (oncoantigens) by three stimulations of peptide-pulsed DCs [30]. We reduced the number of stimulations with peptide-pulsed DCs to one and confirmed that the single stimulation with peptide-pulsed DCs seemed to be sufficient to identify neoantigen-specific TCRs in combination with our TCR sequencing method. As a result, we could significantly shorten the duration to identify neoantigen-reactive TCRs. We demonstrated that TCR sequencing of directly-sorted multimer-positive T cells was able to rapidly identify TCRA and TCRB pairs of dominant clones, using our TCR sequencing method previously developed in various disease conditions including several types of cancer [31–39].

Secondly, our approach use T cells isolated from healthy donors to induce neoantntigen-specific T cells. T cells obtained from healthy donors may have broader T cell repertoire and have a higher chance to induce neoantigen-specific T cells since cancer patients who are heavily treated with anti-cancer drugs, particularly cytotoxic agents, are likely to have severe damage in their immune system and have smaller diversity of T cells [19]. Recently, Lu *et al.* developed a unique approach on TILs co-cultured with tandem minigene-transfected antigen presenting cells to isolate neoantigen-specific TCRs. They performed single-cell RNA sequencing analysis on T cells to identify a pair of TCRA and TCRB sequences of neoantigen-specific T cells [40]. However, this approach requires 3–6 weeks of TIL culture and a large number of TILs need to be examined by single-cell analysis. In our protocol, although peripheral blood T cells of healthy donors might yield the lower proportion of neoantigen-specific T cells compared with TILs, we could identify a dominant pair of TCRA and TCRB of FACS-sorted dextramer-positive T cells. Hence, we believe that our protocol is time-efficient and cost-efficient for establishing neoantigen-specific TCR-engineered T cells. Because a time is very limited for patients with progressive or advanced tumors, how to identify neoantigen-specific TCR sequences is critically important. Since adoptive therapy using expanded TILs, some of which were shown to react on neoantigens, revealed encouraging clinical outcome for melanoma and other types of solid tumor [11, 12], TCR-engineered T cells targeting neoantigens are also considered to be a promising therapy for advanced cancer.

Our data also indicate that the current protocol would benefit from further improvements. In order to avoid autoimmune side effects when using neoantigen-specific TCRs, the TCRs should not recognize the wild-type analogs of mutated antigens as shown in RNF19B_V372L_ found in the ovarian cancer sample. TCRs distinguish neoantigens from its corresponding wild-type peptides if the mutation change the recognition site for the TCR, where T cells can directly interact [41]. The neoantigens with amino acid changes in the HLA anchor position, usually position 2 and 9 for HLA-A*02:01, relies on significant differences in binding affinity between wild-type and mutant peptides [42]. However, both wild-type and mutant of the RNF19B peptides (V372L in position 2 of the epitope) are predicted to have similarly high HLA-binding affinity, and each TCR that is raised against the mutant peptide will probably be reactive against its wild-type counterpart. Our results indicate that a TCR recognizing neoantigen is not always mutated-peptide specific, and we should apply additional selection criterion to demarcate false-positive neoantigens such as RNF19B_V372L_. This would help to clearly earmark the most promising neoantigen epitopes for *in vitro* induction of specific T cells.

In conclusion, the developed protocol facilitates the generation of neoantigen-specific TCRs in a time frame that will allow a patient to benefit from subsequent immunotherapy using TCR-engineered T cells. When considering a small precursor frequency of neoantigen-specific T cells in the blood of third party donors and a judicious selection of neoantigen peptides for stimulation, the protocol will have clear advantages over other strategies that rely on reactivation or induction of T cells in a tolerant repertoire.

## MATERIALS AND METHODS

### Cell lines

C1R (lacking HLA-A and HLA-B, B lymphoblast) were purchased from American Type Culture Collection (Rockville, MD). TE-8 (HLA-A*24:02, esophageal cancer) and TE-11 (HLA-A*24:02, esophageal cancer) cells were provided by the Cell Resource Center for Biomedical Research Institute of Development, Aging and Cancer at Tohoku University. C1R cells stably expressing HLA-A*24:02 (C1R-A24) or HLA-A*02:01 (C1R-A02) were prepared by the transfection of the vectors encoding HLA-A*24:02 or HLA-A*02:01 gene. All cells were cultured under the recommendations of their respective depositors.

### Screening of potential neoantigens

To screen potential neoantigens in TE-8 cancer cell lines having HLA-A*24:02, we used somatic mutation information from the COSMIC database [43]. Among the somatic mutations, we selected non-synonymous mutations, which the expression of mutant RNA were detected at least 1× read using RNA-sequencing bam data obtained from CCLE database, for the further analysis of neoantigen prediction. HLA genotype information was obtained from previous reports [44].

From an ovarian cancer sample having HLA-A*02:01, genomic DNAs and total RNAs were extracted using AllPrep DNA/RNA mini kit (Qiagen, Valencia, CA). As germline control DNAs, genomic DNAs were extracted from PBMCs. Whole-exome libraries were prepared from 1,000 ng of genomic DNAs using SureSelectXT Human All Exon V5 kit (Agilent Technologies, Santa Clara, CA) and the prepared whole-exome libraries were sequenced by 100-bp paired-end reads on HiSeq2500 Sequencer (Illumina, San Diego, CA). Sequence alignment and mutation calling were performed using our in-house pipelines described previously [45]. Briefly, the sequence reads were mapped to the human reference genome GRCh37/hg19 using Burrows-Wheeler Aligner (BWA) (v0.7.10). Possible PCR duplicated reads were removed using Picard tool (http://broadinstitute.github.io/picard/), and reads with a mapping quality of <30 and with mismatches of more than 5% of sequence reads were also excluded. Finally, somatic variants (single nucleotide variations (SNVs) and indels) were called with the following parameters, (i) base quality of ≥15, (ii) sequence depth of ≥ 10, (iii) variant depth of ≥4, (iv) variant frequency in tumor of ≥10%, (v) variant frequency in normal of <2%, and (vi) Fisher *p* value of <0.05. HLA class I genotypes of the tumor were determined by OptiType algorithm using whole-exome sequence data of normal DNAs [45]. Potential neoantigens were predicted for each non-synonymous mutation by testing all 8- to 11-mer peptides harboring the amino acid substitution using NetMHCv3.4 software as described previously [35]. We selected candidates whose predicted binding affinity to HLA-A*24:02 or HLA-A*02:01 was < 500 nM.

The study protocol for this process was approved by the Institutional Review Board of the University of Chicago (approval number 13–0797 and 13–0526) and Saitama Medical University International Medical Center (approval number 15–225). All patients provided written informed consents.

### Induction of neoantigen-specific cytotoxic T lymphocytes (CTLs)

The research protocol for collecting and using PBMCs from healthy donors was approved by the Institutional Review Board of the University of Chicago (approval number 13–0797 and 15–1738). To induce neoantigen-specific CTLs, we first collected PBMCs from healthy donors by means of Vacutainer CPT Cell Preparation Tube (BD Biosciences, San Jose, CA). Neontigen-derived peptides (more than 95% purity) were purchased from Innopep (San Diego, CA). CD8^+^ T cells were separated using Dynabeads CD8 Positive Isolation Kit (Thermo Fisher Scientific, Carlsbad, CA). Monocyte-derived immature DCs were enriched using plastic adherence method, and were cultured in CellGro DC (Cellgenix, Freiburg, Germany) containing 1% Human AB serum (ABS), 1,000 U/mL GM-CSF (R&D Systems, Minneapolis, MN) and 500 U/mL IL-4 (R&D Systems) for 72 h in Primaria 6-well plate (Corning, Inc., Corning, NY) [30]. Then, 10 ng/mL LPS (Sigma-Aldrich, ST. Louis, MO) and 100 U/mL IFN-γ (PeproTech, Rocky Hill, NJ) were added in the culture to induce the maturation of DCs. DCs were pulsed with 20 µg/mL of the respective synthesized peptides for 16 h at 37° C, and treated with 30 µg/mL of mitomycin C (MMC, Sigma-Aldrich) at 37° C for 30 min. Following washing out the residual peptides and MMC, DCs were cultured with autologous CD8^+^ T cells in 0.5 mL of CellGro DC/5% ABS supplemented with 30 ng/mL IL-21 (R&D Systems) on 48-well plate (each well contained 1.0 × 10^5^ peptide-pulsed DCs, 5 × 10^5^ CD8^+^ T cells) on day 1 [46, 47]. Three days later (on days 4), 5 ng/mL IL-7 (R&D Systems) and 5 ng/mL IL-15 (Novoprotein, Summit, NJ) were added in the culture media. On days 6, the cultures were transferred to 12-well plate with CellGro DC/5% ABS containing 5 ng/mL IL-7 and 5 ng/mL IL-15 [48]. On days 8, cultures were supplemented with CellGro DC/5% ABS containing 10 ng/mL IL-7 and 10 ng/mL IL-15. On days 11, neoantigen-specific T cells were assessed using specific dextramer (Immudex, Copenhagen, Denmark) by flow cytometry analysis.

### Flow cytometry and antibodies

Peptide-HLA dextramers labeled with allophycocyanin (APC) were purchased from Immudex. To assess the positivity of neoantigen-specific T cells, the cells were incubated with dextamers for 10 min at room temperature and then treated with fluorescein isothiocyanate (FITC)-conjugated anti-human CD8 antibody (clone HIT8a, BD Biosciences) at 4° C for 20 min. To examine the expression of CD137, TCR-engineered T cells were incubated with anti-human CD137 antibody (clone 4B4-1, Miltenyi Biotec, Bergisch Gladbach, Germany). To confirm the expression of HLA-A24, TE-8 and TE-11 cells were stained with anti-human HLA-A24 antibody (clone 17A10, MBL, Woburn, MA). Fluorescence was quantified by flow cytometry (FACS LSRII; Becton Dickinson, San Jose, CA). Data analysis was performed using Flow Jo software (Treestar, Ashland, OR).

### TCR sequencing

We determined TCR sequences using the method we previously developed [35, 49]. In brief, we extracted total RNAs from FACS-sorted dextramer-positive T cells. cDNAs with common 5′-RACE adapter were synthesized using SMART library construction kit (Clontech, Mountain View, CA). The fusion PCR was performed to amplify TCRA and TCRB cDNAs using a forward primer corresponding to the SMART adapter sequence and reverse primers corresponding to the constant region of each of TCRA and TCRB. After adding the Illumina index sequences with barcode using the Nextera Index kit (Illumina, San Diego, CA), the prepared libraries were sequenced by 300-bp paired-end reads on the MiSeq (Illumina). Obtained sequence reads were analyzed using Tcrip software [49].

### TCR-engineered T cells

Both TCRA and TCRB sequences were codon-optimized and cloned into a retroviral vector, pMP71-PRE as described previously [50]. pMP71-PRE lacks its own replicating ability. To improve TCR surface expression, we employed constant regions of mouse TCR [51]. Transient retroviral supernatants were generated and PBMCs from donors were transduced as described previously [52].

To examine the cytotoxic activity by DPY19L4_L143F_ TCR-engineered T cells, we enriched TCR-engineered T cells using the staining with APC-conjugated anti-mouse TCR beta monoclonal antibody (H57-597, eBioscience, San Diego, CA) at 1:2000 (0.1 µg/mL) followed by the incubation with anti-APC microbeads (Miltenyi Biotec) according to the manufacturer’s instructions.

### ELISPOT and ELISA assays

ELISPOT assay was performed using Human IFN-γ ELISpot^PRO^ kit (MABTECH, Cincinnati, OH) according to the manufacturer’s instruction. Briefly, antigen presenting cells were pulsed with respective peptides at 37° C for 20 h, and the residual peptides that did not bind to cells were washed out to prepare peptide-pulsed cells as the stimulator cells. T cells were pre-treated with IL-2 (35 U/mL) for 16 h and then co-incubated with peptide-pulsed stimulator cells (2 × 10^4^ cells/well) at 37° C for 20 h in 96-well plate. The supernatant was transferred into a new 96-well plate for ELISA assay. Spots were captured and analyzed by an automated ELISPOT reader, ImmunoSPOT S4 (Cellular Technology Ltd, Shaker Heights, OH) and the ImmunoSpot Professional Software package, Version 5.1 (Cellular Technology Ltd). To measure the secreted cytokine levels in the supernatant, we used OptEIA Human IFN-γ ELISA set (BD Biosciences), Human Granzyme B ELISA development kit (MABTECH) and read absorbance in a microplate reader at 450/570 nm.

### Evaluation of cytotoxic activities of CTLs against cancer cells by time-lapse recording

TCR-engineered T cells were pre-treated with IL-2 (100 U/mL) for 16 h. Target cells were pre-treated with IFN-γ (100 U/mL) for 48 h before experiments. The target cells were incubated with 1 µg/mL of Calcein AM (Dojindo, Kumamoto, Japan) for 30 min. After 3-time wash by PBS, 2 × 10^4^ of target cells were mixed with 4 × 10^5^ TCR-engineered T cells into Lab-Tek Chamber Slide Cover Glass Slide Sterile 16 Well (Thermo Fisher Scientific). We used automated imaging system with high-resolution (EVOS FL Auto 2 Imaging System, Thermo Fisher Scientific).

### Statistical analysis

The student’s *t* test was performed for comparison of the number of IFN-γ positive spots, the concentration of IFN-γ and granzyme B between HLA-A*24:02- or mock-transfected TE-8 cells when co-cultured with DPY19L4_L143F_ TCR-engineered T cells. Statistical analyses were done using GraphPad Prism version 6.0 (GraphPad software, La Jolla, CA). *P* value of <0.05 was considered to be statistically significant.

## SUPPLEMENTARY MATERIALS FIGURES, TABLES AND VIDEOS













## Abbreviations

CDR3: complementarity determining region 3
HLA: human leucocyte antigen
PBMCs: peripheral blood mononuclear cells
TCR: T cell receptor
TCRA: T cell receptor alpha
TCRB: T cell receptor beta
TILs: tumor-infiltrating lymphocytes
