# Supplementary material for: Effective screening of T cells recognizing neoantigens and construction of T-cell receptor-engineered T cells
Source: Oncotarget. 2018 Jan 13;9(13):11009–19. doi: 10.18632/oncotarget.24232 (PMC5834292; doi:10.18632/oncotarget.24232)
Supplement: Supplementary file 1 [file oncotarget-09-11009-s001.pdf]

Effective screening of T cells recognizing neoantigens and construction of T-cell receptor-engineered T cells

SUPPLEMENTARY MATERIALS

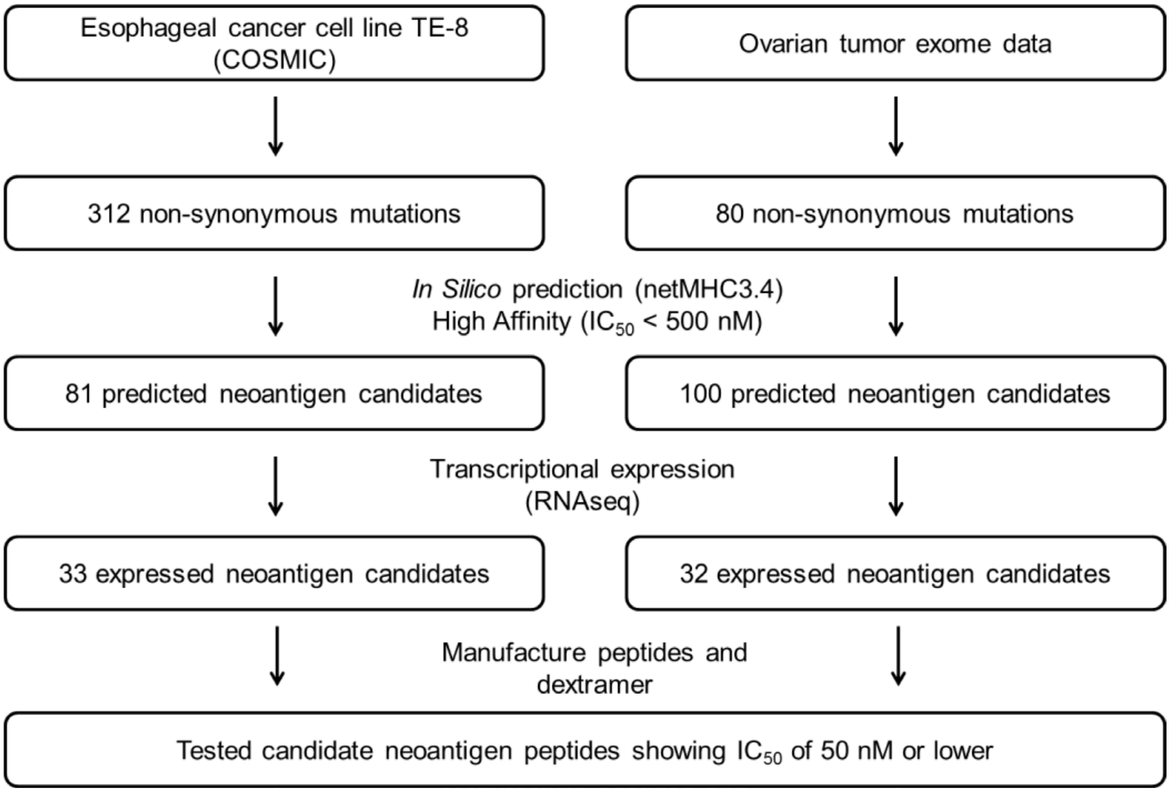

Supplementary Figure 1: Neoantigen selection workflow. Schema diagram of selection of possible neoantigen candidates from TE-8 cell line with the COSMIC mutation data (left) and an ovarian cancer exome data (right).

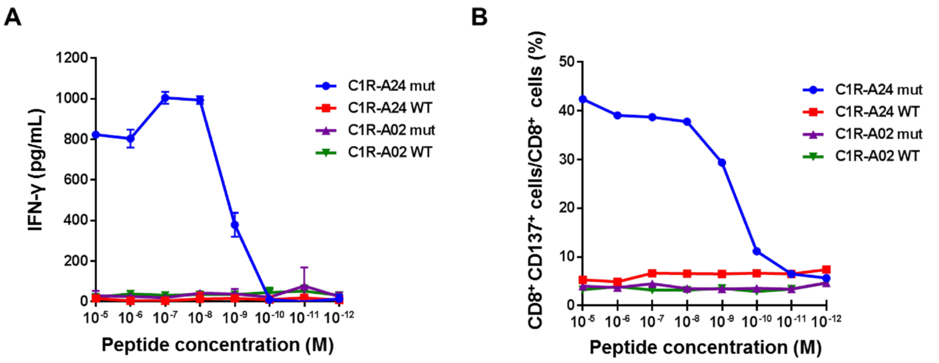

Supplementary Figure 2: *In vitro* analysis of DPY19L4$_{L143F}$ TCR-engineered T cells. (**A**) IFN-γ ELISA assay and (**B**) CD137 assay on DPY19L4$_{L143F}$ TCR-engineered T cells co-cultured with C1R-A24/A02 cells loaded with graded amounts of peptide.

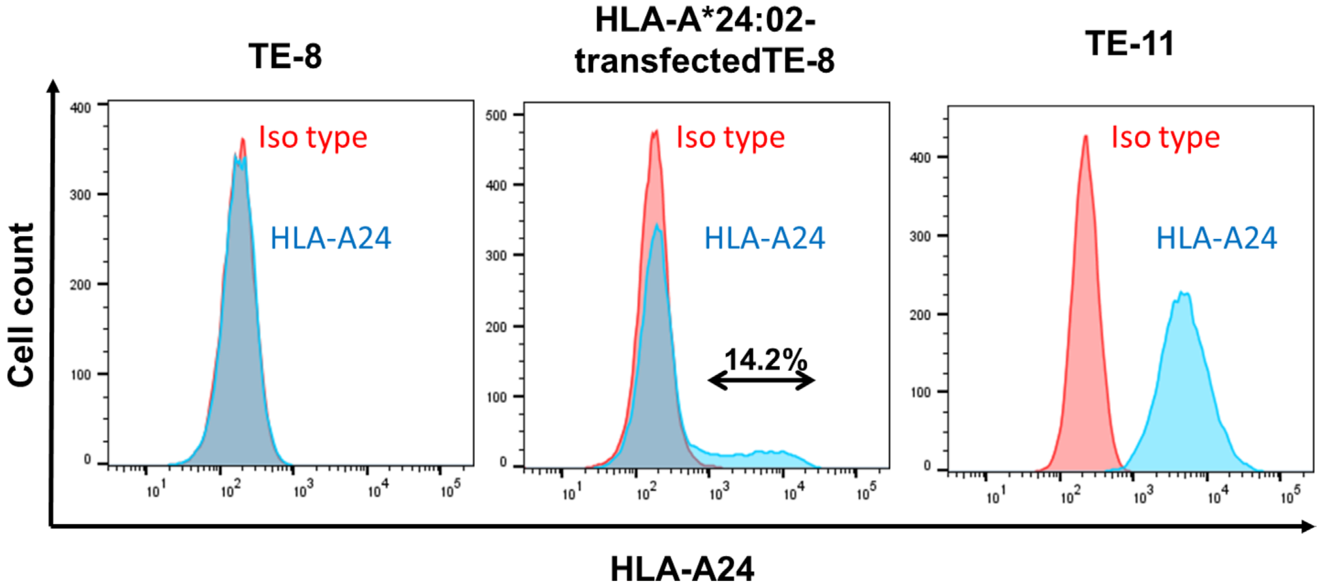

Supplementary Figure 3: Flow cytometry analysis (HLA-A24 staining). Flow cytometry figures are the representative of HLA-A24 staining for TE-8, HLA-A*24:02-transfected TE-8 and TE-11 cells.

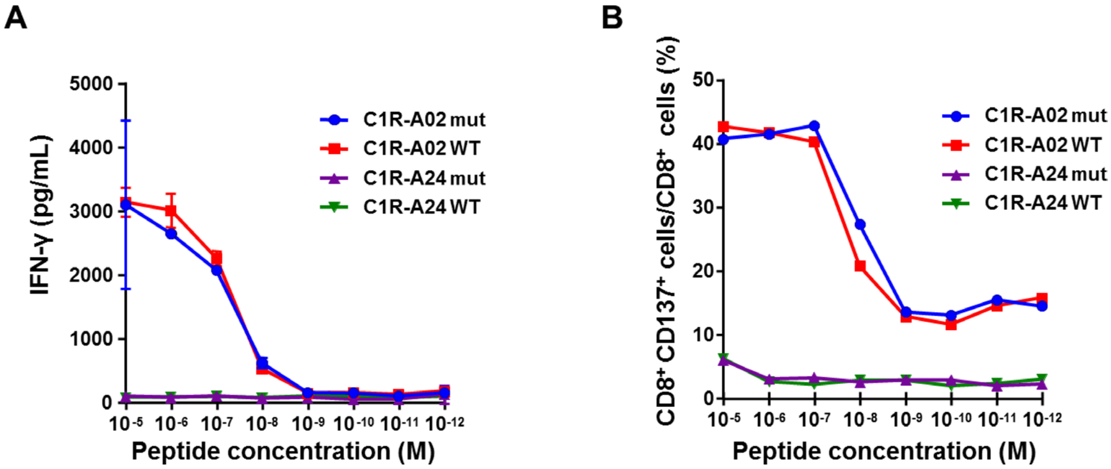

Supplementary Figure 4: *In vitro* analysis of RNF19B$_{V372L}$ TCR-engineered T cells. (**A**) IFN-γ ELISA assay and (**B**) CD137 assay on RNF19B$_{V372L}$ TCR-engineered T cells co-cultured with C1R-A24/A02 cells loaded with graded amounts of peptide.

Supplementary Table 1: List of neoantigen peptides expressed and predicted to HLA-A24:02 with affinities of <500 nM in TE-8 cell line. See Supplementary_Table_1

Supplementary Table 2: List of detected mutations in ovarian cancer patient. See Supplementary_Table_2

Supplementary Table 3: List of neoantigen peptides expressed and predicted to HLA-A02:01 with affinities of <500 nM in ovarian cancer patient. See Supplementary_Table_3

Supplementary Table 4: Sequences of dominant TCRs for neoantigen-reactive T cells

	TRAV	TRAJ	CDR3	TRBV	TRBJ	CDR3
DPY19L4$_{L143F}$ TCR	TRAV3	TRAJ41	CAVRGTSGYALNF	TRBV25-1	TRBJ1-2	CATSFAPGQGEHGYTF
RNF19B$_{V372L}$ TCR	TRAV12-3	TRAJ36	CAMSPLLETGANNLFF	TRBV28	TRBJ1-2	CASSTSTGQGWHYGYTF

Supplementary Movie 1: Cytotoxic activity of DPY19L4$_{L143F}$TCR-engineered T cells co-cultured with mutant peptide-pulsed TE-11 cells. See Supplementary_Movie_1

Supplementary Movie 2: Cytotoxic activity of DPY19L4$_{L143F}$ TCR-engineered T cells co-cultured with peptide non-pulsed TE-11 cells. See Supplementary_Movie_2
